# Effects of the Ergot of Rye on the Parturient Female and Her Offspring

**Published:** 1848-01

**Authors:** 


*Effects of the Ergot of Rye on the Parturient female and her offspring.*—With the view of throwing some further light on the action and effect of the ergot, Dr. Samuel L. Hardy, of the Dublin Lying-in Hospital, has kept accurate notes of a large number of cases, in which this drug has been administered during parturition. Several of his observations are of considerable value.

*Time at which the action of the ergot on the uterus commences.*—From comparing tables which the author has drawn up, it appears that, in some cases, ergot acts on the uterus, so soon as seven minutes after its administration, whilst in others, a much longer period of time is required; but in the generality, from about ten to fifteen minutes may be stated as the average. In those cases where the children have been expelled alive, Dr. Hardy has always observed the action of ergot on the uterus, to commence within twenty-five minutes. On the other hand, when a longer period than that elapses before the uterus takes on action, the use of instruments has been necessary to perfect the delivery, or the children have been dead born. In some instances, the ergot has produced in the uterus a kind of tonic contraction, without any effective expelling pains. In accordance with what has been observed by others, the author has noticed that, in those cases where the ergot has acted beneficially, its exhibition is followed by strong expulsive pains, which gradually increase in frequency, so that, in fact, they may be said to run into each other, there being no distinct interval between them.

*Effect on the Pulse.*—In nineteen cases of those which Dr. Hardy has recorded, there was a marked diminution in the frequency of the mother's pulse, following the administration of ergot, and this effect generally began to take place from about fifteen minutes to half an hour. In all these instances where the depression of the pulse occurred, the fœtal heart underwent a similar change. Hence the author is led to inquire, is ergot a safe remedy in a case where the woman is greatly reduced by hemorrhage arising from relaxation of the uterus after delivery? He mentions a case bearing upon this point, where a draining had continued for several hours after the expulsion of the placenta, by which the patient was greatly weakened; the usual dose of powdered ergot was given, and was followed almost immediately after by most alarming depression, requiring the administration of the most powerful stimulants. In several of the cases the depressed state of the circulation continued for several days, notwithstanding, in some instances, inflammation of the uterus followed delivery; and the uterine tumours not unfrequently remained much larger than natural, even where there was no reason to suspect the presence of inflammation of that organ.

*Effects of Ergot on the Fœtal Heart.*—The effect of ergot on the fœtal heart, is still more remarkable than on the maternal pulse, and, in a practical point of view, deserves a much more serious investigation.

In a great majority of the author's cases, a diminution in the fœtal heart's pulsations followed the administration of ergot. The period at which this effect begins to be produced, varies from about fifteen minutes to half an hour, sometimes a little sooner, and occasionally at a later period. The most common effect, and usually the first the author has observed, is a diminution in the frequency of the pulsations; this is succeeded, after some time, by an irregularity in its beats, which irregularity continues, more or less, until the sounds intermit, and at length, after a variable period, become quite inaudible. Dr. Hardy has been led by his observations to the practical inference that, in those cases where the number of the fœtal heart's pulsations have been steadily reduced below 110, and at the same time, with intermissions, the child will be rarely, if ever saved, although its delivery should be effected with the greatest possible speed. But the mere depression of the fœtal heart below 110, without intermission, is not, in itself, sufficient to cause this result, as instances have occurred where the number of pulsations has been still more reduced, (in one case as low as 56,) and yet by speedy delivery, and adoption of the usual remedies, the children have been saved. But in none of these cases was there a steady, distinct, and well-marked intermission. The knowledge of these facts points out the necessity of watching closely the state of the fœtal heart, after the administration of ergot, as delay beyond a particular time cannot be allowed with impunity to the life of a child. Should the case, in other respects, be eligible for the application of the forceps or vectis, in order to save the child, it must be had recourse to within a certain period, which can only be known by the careful use of the stethoscope. The author's observation fully coincide with those of Dr. Beatty, who fixes the limit beyond which the child will rarely be born alive, at two hours. To this rule he has met with but three exceptions. But death of the fœtus may occur long before the expiration of two hours. In two instances, the children were lost, although only twenty minutes in one, and twenty-five in the other, had passed from the administration of the ergot to their expulsion. In these instances, the depressing effects of the ergot are so great, that frequently after births, a considerable time elapses before the children can be perfectly restored; and Dr. Hardy has observed, that infants born in a weak state, where no ergot was given to cause their expulsion, have been restored to animation with must less difficulty, than in those cases in which this medicine was administered during labour. Hemorrhage, after the birth of the child, is an occurrence the author has never met with in any case where the uterus was sensibly affected by the ergot during labour.

With some few exceptions, the women had generally good recoveries. Of those who were attacked with inflammation, all recovered but two. One was a case of retained placenta where the hand was introduced; this patient died of uterine phlebitis. In the second, there was inflammation of the peritoneum and uterus.

The children who were born alive, all, with one exception, did well. In this case, delivery was effected by the forceps, as the fœtal heart had fallen so low as 100 from the effect of the ergot. This statement refers only to those cases where complete restoration was accomplished after delivery.—*Dub. Jour. Med. Sci., from St. Louis Med. and Sur. Jour.*

